# Electrophysiological findings for peripheral nerves of lower limb in women with stress urinary incontinence and non-specific low back pain

**DOI:** 10.1038/s41598-025-06202-7

**Published:** 2025-06-20

**Authors:** Doaa A. Abdel Hady, Lama Eid, Mina George, Osama M. Abdel Raheem

**Affiliations:** 1 Department of Physical Therapy for Women’s Health, Faculty of Physical Therapy, Deraya University, El Minya, Egypt; 2 Faculty of Physical Therapy, Deraya University, El Minya, Egypt; 3 Department of Physical Therapy for Neurology, Faculty of Physical Therapy, Deraya University, El Minya, Egypt

**Keywords:** Stress urinary incontinence (SUI), Non-specific low back pain, Electrophysiological finding, Tibial nerve, Peroneal nerve, Sural nerve, Health care, Urology

## Abstract

Stress urinary incontinence (SUI) and nonspecific low back pain (NSLBP) are prevalent conditions that significantly affect women’s quality of life. Recent studies have identified a connection between these conditions and dysfunction in the peripheral nerves of the lower limb. This study aims to compile existing knowledge on the electrophysiological findings in the peripheral nerves of the lower limb in women experiencing SUI and NSLBP. This was a prospective observational study involving fifty healthy women and women suffering from SUI and NSLBP. The participants were aged between 25 and 35 and had a body mass index (BMI) ranging from 20 to 24. The primary outcome measures focused on evaluating lower-limb peripheral nerves in women with SUI and NSLBP. There was an increase in both distal and proximal latencies of the tibial nerve, along with a significant decrease in distal and proximal amplitudes and nerve conduction velocity (NCV) in group A when compared to group B (*p* < 0.001). For the peroneal nerve, group A showed a significantly higher distal latency and a significantly lower NCV (*p* < 0.001), while no significant differences were observed in proximal latency or the proximal and distal amplitudes between the two groups (*p* > 0.05). Regarding the sural nerve, group A had significantly higher onset latency and lower amplitude and NCV compared to group B (*p* < 0.01). These findings reveal statistically significant nerve conduction abnormalities in tibial, peroneal, and sural nerve conduction in women suffering from stress urinary incontinence (SUI) and non-specific low back pain. The tibial nerve displayed extended distal and proximal latencies, diminished amplitudes, and lower nerve conduction velocity (NCV). The peroneal nerve showed prolonged distal latency and a reduced NCV, while the sural nerve had an extended onset latency, decreased amplitude, and slower NCV. The tibial nerve showed the most changes, including extended latencies and lower conduction velocity, implying both axonal and demyelinating involvement. These changes, supported by large effect sizes, high thresholds commonly associated with clinically meaningful decline, and may have functional consequences.

## Introduction

Urinary incontinence (UI) is defined as the involuntary loss of urine that poses a social or hygienic problem. Common types include stress urinary incontinence (SUI) and urge urinary incontinence (UUI)^1,2^. The prevalence of UI can range from 3 to 55%, increasing with age, particularly in women over 80^2^.

SUI is defined as “the complaint of any involuntary loss of urine on effort or physical exertion (e.g., sporting activities) or on sneezing or coughing”^3^. Reportedly increasing frequency is elements including age, pregnancy, childbirth, and hormone-related disorders^4^. Low back pain (LBP) is the most common musculoskeletal disorder. It has been described as either with or without leg pain as pain, discomfort, muscle tension, or stiffness limited below the costal margin over the inferior gluteal folds^4–8^.

A positive relationship between LBP and SUI was reported in middle-aged women, with SUI potentially predisposing individuals to LBP^4^. Additionally, women with UI demonstrated a significantly higher relationship with LBP and disability^4^. These findings indicate a potential connection between LBP and UI.

Although the exact mechanism in the growth of NSLBP is unknown, it could be connected with changes in the trunk’s muscle control, especially diminished postural activity of the diaphragm, transversus abdominis, and pelvic floor muscles (PFMs), which seem to be related with impairment in the mechanical support of the spine, favoring the start of LBP^9,10^. PFM could impact on Quality of life^11^. These findings suggest that sagittal spinal alignment and lumbopelvic mobility should be considered in the management of UI and related LBP in women. As I mentioned earlier, many studies discussed the relationship between lumbar and pelvis biomechanics and LBP in women with UI^12^.

This study hypothesizes that women who have SUI and NSLBP are likely to show notable changes in the conduction parameters of their tibial, peroneal, and sural nerves when compared to those who don’t have these issues. These alterations could point to some underlying neuromuscular problems that might be playing a role in the symptoms of both SUI and NSLBP. This study aims to find out if there is a relationship between the peripheral nerves of the lower limb in women experiencing SUI and NSLBP.

## Materials and methods

### Study design

Cross-sectional study according to the STROBE declaration^13^. The Helsinki Declaration and human experimentation guidelines were checked, and ethical approval was obtained from Deraya University’s Ethics Committee (approval number DCSR-07024-13). Participants were informed and given written consent before being included in the study. Participants received thorough information regarding the study’s goals, methods, possible risks, and advantages. Joining the study was completely voluntary, and anyone could opt out at any moment without facing any repercussions. We made sure to protect confidentiality and anonymity throughout the research, using the data exclusively for research purposes. It was done from August 1, 2024, to January 1, 2025.

### Participants and recruitment

**Fig. 1:**
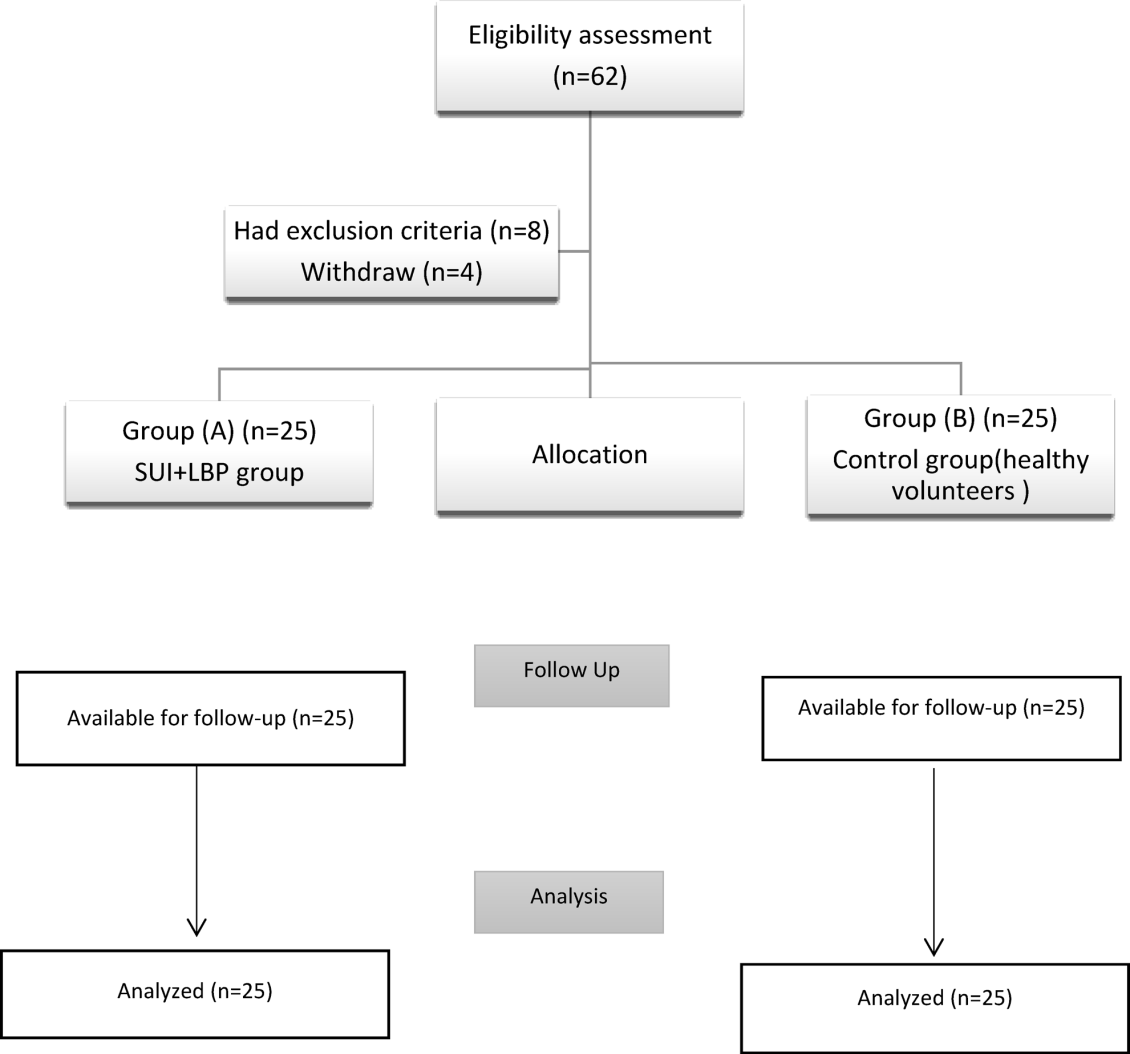
Flow chart of the study.

50 women were recruited from the gynecological outpatient clinic based on the orthopedic specialist and gynecologist’s diagnosis and recommendation. Twenty-five participants from each group. Women with SUI and non-specific LBP were assigned to "Group A," and healthy volunteers were assigned to the control group “Group B" (Fig. 1).

### Inclusion and exclusion criteria

#### Inclusion criteria

Fifty married women aged 25–35, with a BMI between 20 and 24, who experienced a normal vaginal delivery with no more than two deliveries, were selected to participate. 25 women were healthy volunteers, and based on the gynecologist’s advice, 25 women were found to have the condition of nonspecific LBP and mild and moderate SUI. Twenty-five healthy women were randomly selected for the study from the urogynecology center clinic. By using the Urinary Incontinence Short Form on the Incontinence Questionnaire, a score of ≥ 3 was required to be eligible; those who did not achieve this value were not included. The women suffering from SUI, in detail, have reported that they have improvements in many ways, for example, curing leaks when coughing or sneezing, if any, besides, no leaks with physical activities or exercises, or even no leaks when lifting heavy objects. As a minimal participation requirement, women had to choose "I leak when I am physically active/exercising." To find out how much the pelvic floor is implicated in MUI and how severe the problems are for the patients, the Medical, Epidemiologic, and Social Aspects of Aging Questionnaire (MESA) was used. Furthermore, two additional questions were inquired about, such as if the women had experienced LBP (yes/no), where the LBP happened in the last six months, and whether they have a VAS score of 4–8 on moderate pain.

#### Exclusion criteria

Past pelvic surgery, ongoing or planned pregnancy, musculoskeletal injuries within the past six months, uninterrupted injury affecting the training program, pelvic organ prolapse (POP), examined utilizing the POP-Q system following the recommendations of the International Continence Society^14^, type, an evaluation of the presence of at least one of the diseases from the group of mental and neurological disorders was carried out; along with the exclusion of unmarried women and those who have the alternative type of UI, women with a history of cesarean sections, and those having the problem of the prolapse of the reproductive organ. Moreover, using estrogen medication and having high blood pressure and cardiac weakness were the limitations of the exclusion criteria. Women were not excluded from the study if they suffered from conditions such as urinary tract infections, autonomic disorders, neurological problems, cardiovascular diseases, and pudendal nerve injuries, and were cooperative with the screening session of the study.

### Data collection

Before being included in the study.

#### The medical, epidemiological, and social aspects of aging questionnaire

The medical, epidemiological, and Social Aspects of Aging questionnaire has been developed and validated to measure the urgency- or stress-predominant component of MUI and the symptom severity. The self-administered MESA questionnaire contains nine questions on stress incontinence and six on urge incontinence. Higher scores reflect higher symptom frequencies. The four response categories range from “never” (0 points) to “often” (3 points). The stress score, the urge score, the stress index, and the urge index were calculated by dividing the actual score of each category by the maximum possible total: the stress score (maximum = 27 points), the urge score (maximum = 18 points), the stress index, and the urge index. If the stress index is higher than the urge index, MUI is defined as stress-predominant. The total score of stress is 27; for determining the severity of incontinence, it is divided into three categories, three degrees: scores ranging from 1 to 9 are assigned as mild, from 10 to 18 as moderate, and from 19 to 27 as severe^15^.

#### ICIQ UI SF

The primary outcome was defined as a change in the total score of ICIQ-UI-SF^16^. ICIQ-UI-SF represents a valid and reliable measure for the frequency, amount of leakage, impact on daily life, and type of UI. This questionnaire has four questions; three of them give a total score of 0 to 21, while the fourth question allows classification of the UI type. An MCID of 2.5 in the ICIQ-UI-SF total score has been determined^17^.

“Urine leakage frequency is measured and ranges from 0 (never) to 5 (always). Leakage Amount: Calculates the average amount of urine lost, with a score ranging from 0 (none) to 6 (a lot). Overall Impact on Daily Life: Scores range from 0 (not at all) to 10 (a great deal), indicating how much urine leakage disrupts daily activities. Higher scores indicate more severe incontinence. Slight: 1–5, Moderate: 6–12, Severe: 13–18, and Very Severe: 19–21 are the severity levels^18^.

#### The visual analogue scale (VAS)

It is a validated subjective measure for acute and chronic pain. Scores are recorded by making a handwritten mark on a 10-cm line representing a continuum between “no pain” and "worst pain.” To quantify the pain intensity on a scale from 0 to 10, patients mark a point along the line that represents their perceived level of pain^14^.

#### EMG assessment

All EMG tests were conducted by a neurologist, and the assessors were kept unaware of which participants belonged to which groups. The evaluation procedures involved a nerve conduction study using the Neuro Pack M1 Ep/EMG measuring system. This system was utilized to conduct sensory and motor conduction studies on all peripheral nerves of the lower limb, including the tibial, peroneal, and sural sensory nerves. The study was conducted at the Faculty of Physical Therapy, Deraya University.

Motor and sensory nerve conduction studies (NCS) were performed unilaterally (left side) on each subject that is to be oriented on the following three parameters: nerve conduction velocity (NCV), amplitude, and distal delay. The recordings were carried out by correct distance measurements, well-defined, artifact-free responses, and at a temperature of 32–34 °C using air warming systems. All nerve responses are recorded on 4 × 7 mm surface silver/silver chloride discs. The values of repeated nerve conduction velocities (CVs) and amplitudes were averaged from two nerve conduction investigations^19^. The amplitude of the compound muscle action potential (CMAP) was followed from the baseline to the negative peak, and the distal motor latency (DML) was the time from the stimulus to the first peak of CMAP from the baseline. Sensory NCS was performed using the antidromic examination^16^. The way from the stimulus to the first negative deflection from baseline for biphasic or the first positive peak for triphasic was considered the onset delay for the sensory nerve action potentials (SNAP) checks. The distance between the baseline and the negative peak was the amplitude that was being determined. To control for this, NCS reference values (the ones people without health problems have) from our lab group were used. The patients were positioned side-lying with the leg of interest on top during the nerve conduction tests^16^. The way from the stimulus to the first negative deflection from baseline for biphasic or the first positive peak for triphasic was considered the onset delay for the sensory nerve action potentials (SNAP) checks. The distance between the baseline and the negative peak was the amplitude that was being determined. To control for this, NCS reference values (the ones people without health problems have) from our lab group were used. The patients were positioned side-lying with the leg of interest on top during the nerve conduction tests^16^.

Motor nerve conduction studies (MCS) were carried out by evaluating the tibial nerve, which was done by putting the active recording electrode on the abductor hallucis brevis muscle and the reference electrode on the MTPJ of the great toe. Two sites were stimulated: one distal to the medial malleolus (9 cm distance) and one proximal at the middle of the popliteal fossa (over the popliteal pulse)^20^. The peroneal nerve was assessed by using an active electrode on the extensor digitorum brevis muscle and the reference electrode on the MTPJ of the little toe. Stimulation was done on one side close to the ankle, lateral to the tibialis anterior tendon (9 cm distance), and on the other side proximally below the fibular head (2 fingers below the fibular head), with the ground electrode placed between the stimulator and the active electrode^20^.

The sensory nerve conduction studies (SCS) were performed by putting the active recording electrode posterior to the lateral malleolus and the reference electrode 3–4 cm distal to the active site. Stimulation has been performed at this point posterolateral to the calf (14 cm distance) with the ground electrode placed between the stimulator and the active electrode^19^.

### Sample size

Sample size calculation was performed using G*POWER statistical software (version 3.1.9.2; Franz Faul, Universität Kiel, Germany) based on data of tibial NCV derived from a pilot study conducted on 5 subjects in each group, and revealed that the required sample size for this study was 50 subjects. The calculation was made with α = 0.05, power = 80%, effect size = 0.82, and allocation ratio 1:1.

### Statistical analysis

An unpaired t-test was conducted to compare age and BMI between groups, while a Mann–Whitney test was used to compare parity as discrete data. The normal distribution of data was checked using the Shapiro–Wilk test. Levene’s test for homogeneity of variances was conducted to test the homogeneity between groups. An unpaired t-test was conducted to compare latency, amplitude, and NCV of tibial, peroneal, and sural nerves between groups. The level of significance for all statistical tests was set at *p* < 0.05. All statistical measures were performed through the Statistical Package for Social Sciences (SPSS) version 25 for Windows.

## Results

### Subject characteristics

Subjects’ characteristics are demonstrated in Table 1. There was no significant difference between groups in age, BMI, and parity (*p* > 0.05).

**Table 1:** Basic characteristics of participants.

	Group A	Group B	t-value	*p* value
	Mean ± SD	Mean ± SD		
Age (years)	29.72 ± 3.10	30.68 ± 2.82	− 1.14	0.26
BMI (kg/m^2^)	23.39 ± 1.11	23.84 ± 1.20	− 1.36	0.18
Parity, median (IQR)	1 (2–1)	1 (2–1)	(U = 263)	0.28

SD, standard deviation; IQR, Interquartile range; U-value: Mann–Whitney test value; *p* value, probability value.

### Comparison of latency, amplitude, and conduction velocity of tibial, peroneal, and sural nerves between groups

There was a significant increase in tibial nerve distal and proximal latencies and a significant decrease in distal and proximal amplitudes and NCV in group A compared to group B (*p* < 0.001).

In the peroneal nerve, distal latency was significantly higher in group A, and NCV was significantly lower in group A (*p* < 0.001), while there were no significant differences in proximal latency and proximal and distal amplitudes between groups (*p* > 0.05).

For the sural nerve, group A exhibited significantly higher onset latency and lower amplitude and NCV compared to group B (*p* < 0.01) (Table 2).

**Table 2:** Comparison of Distal latency, amplitude, and conduction velocity of tibial, peroneal, and sural nerves between groups A and B.

	Group A	Group B		95% CI		t- value	*p *value	Effect size
	Mean ± SD	Mean ± SD	MD	Lower limit	Upper limit			
Tibial nerve								
Distal latency (msec)	5.35 ± 0.53	4.13 ± 0.64	1.22	0.88	1.55	7.33	0.001	2.08
Proximal latency (msec)	13.41 ± 1.08	12.20 ± 0.84	1.21	0.66	1.76	4.42	0.001	1.25
Distal amplitude (uV)	9.82 ± 1.86	11.57 ± 1.20	− 1.75	− 2.64	− 0.86	− 3.95	0.001	1.12
Proximal amplitude (uV)	7.89 ± 2.56	9.97 ± 1.51	− 2.08	− 3.27	− 0.88	− 3.49	0.001	0.99
NCV (m/sec)	46.86 ± 2.55	50.92 ± 2.50	− 4.06	− 5.49	− 2.62	− 5.67	0.001	1.60
Peroneal nerve								
Distal latency (msec)	2.87 ± 0.49	2.44 ± 0.40	0.43	0.18	0.68	3.41	0.001	0.96
Proximal latency (msec)	10.64 ± 1.17	10.45 ± 0.92	0.19	− 0.41	0.79	0.65	0.52	0.18
Distal amplitude (uV)	4.42 ± 2.33	5.22 ± 2.38	− 0.8	− 2.15	0.53	− 1.21	0.23	0.34
Proximal amplitude (uV)	3.84 ± 2.02	3.90 ± 2.17	− 0.06	− 1.24	1.14	− 0.08	0.93	0.02
NCV (m/sec)	47.16 ± 3.44	51.65 ± 5.56	− 4.49	− 7.12	− 1.86	− 3.43	0.001	0.97
Sural nerve								
Onset latency (msec)	2.24 ± 0.45	1.85 ± 0.29	0.39	0.17	0.60	3.57	0.001	1.01
Amplitude (uV)	24.38 ± 7.40	30.15 ± 6.39	− 5.77	− 9.70	− 1.83	− 2.94	0.005	0.83
NCV (m/sec)	57.81 ± 15.97	55.41 ± 4.16	− 3.93	− 6.65	− 1.22	− 2.91	0.005	0.82

SD, standard deviation; MD, mean difference; CI, confidence interval; *p* value, probability.

In Group A, NCV values are often within the reference range but are reduced significantly compared to group B**.** Effect sizes were large (Cohen’s d > 0.8) for most parameters, especially tibial latency (d = 2.08) and NCV (d = 1.60). These results support a diagnosis of subclinical peripheral nerve problems in Group A, with both motor and sensory nerve involvement (Table 3).

**Table 3:** Diagnostic comparison and pathophysiological interpretation.

Nerve	Parameter	Group A mean	Diagnostic threshold	Meets neuropathy criteria	Pathology
Tibial	NCV (m/s)	46.86	< 40–41 m/s (motor)	No—borderline	Mixed (demyelination + axonal)
	Distal amplitude (µV)	9.82	< 6 µV	No	Mild axonal reduction
Peroneal	NCV (m/s)	47.16	< 43 m/s (motor)	No	Demyelinating
	Distal amplitude (µV)	4.42	< 2 µV	No	Preserved axon, slowed conduction
Sural	NCV (m/s)	57.81	< 40 m/s (sensory)	No	Normal
	Amplitude (µV)	24.38	< 6 µV	No	Axonal involvement

## Discussion

Numerous studies have explored the relationship between low back pain (LBP), stress urinary incontinence (SUI), and pelvic floor muscle (PFM) function in women. Although there is limited research on the peripheral nerves of the lower leg in this area, the following papers offer valuable insights into how these conditions are interconnected.

This study showed an increase in both distal and proximal latencies of the tibial nerve, along with a significant decrease in distal and proximal amplitudes and nerve conduction velocity (NCV) in group A when compared to group B (*p* < 0.001). For the peroneal nerve, group A showed a significantly higher distal latency and a significantly lower NCV (*p* < 0.001), while no significant differences were observed in proximal latency or the proximal and distal amplitudes between the two groups (*p* > 0.05). Regarding the sural nerve, group A had significantly higher onset latency and lower amplitude and NCV compared to group B (*p* < 0.01). Group A nerve conduction values are often within the reference range but are reduced significantly compared to group B**.** Effect sizes were large (Cohen’s d > 0.8) for most parameters, especially tibial latency (d = 2.08) and NCV (d = 1.60).

Detrusor muscle contraction, sphincter control, and sensory feedback are all governed by a complex neural network that includes the pelvic, pudendal, and hypogastric nerves. To start urinating, the parasympathetic (S2–S4) pelvic nerve stimulates the contraction of the detrusor muscle. The sympathetic hypogastric nerve (T10–L2) contracts the internal urethral sphincter and relaxes the detrusor muscle to permit bladder filling. The external urethral sphincter is controlled by the pudendal nerve (Somatic: S2–S4), which permits voluntary control over urination. Feedback on bladder fullness is provided by sensory nerves^21^.

The pudendal nerve, the primary nerve that governs the PFM it innervates, includes the external urethral sphincter, coccygeus, levator ani, and external anal sphincter^22^. UI can be treated by stimulating the tibial and peroneal nerves, which are indirectly connected to the bladder and pelvis through the autonomic nervous system^23^.

Another Explanation for this finding is that, together with several other nerves and vessels, the sciatic nerve leaves the pelvis through the larger sciatic foramen located beneath the piriformis muscle. It descends through the posterior thigh, passing superficially to the adductor magnus and short head of the biceps femoris and deep to the long head of the biceps femoris. It divides into the common peroneal nerve, which travels through the lateral and anterior leg and foot, and the tibial nerve, which continues through the back of the leg and foot before it reaches the popliteal fossa^24^. It can be supported by Research that has explored activating the tibial, parasacral stimulation, pudendal stimulation, and peroneal nerves, Neuromodulation has been investigated as a treatment for SUI^25–27^.

The tibial and peroneal nerves, both terminal branches of the sciatic nerve, arise from the lumbosacral plexus (L4-S3), which also provides rise to the pudendal nerve, the primary motor and sensory supply to the PFM. These nerves have overlapping spinal roots, particularly S2 and S4, which are important for synchronizing PFM and lower limb motor processes. As a result, anomalies in tibial or peroneal nerve conduction may reflect broader subclinical disorders within the lumbosacral system, including neural control over PFMs^28–31^.

### Strengths and limitations

This study has limitations. These findings reveal a notable relationship between SUI, NSLBP, and peripheral nerve dysfunction. However, causality cannot be determined due to the observational nature of the study and the narrow inclusion criteria, as the study focused only on married women aged 25–35 with a BMI of 20–24. It would be great for future research to look into whether focusing on these neural pathways could lead to better clinical results and improved treatments. And should include a more diverse sample. Another important limitation was the use of only subjective tools, such as ICIQ UI SF, MESA, and VAS, for the assessment of the participants before being included in the trial. All electrophysiological assessments were conducted by a single blinded assessor, while this approach minimizes inter-rater variability. Currently, it has been used in clinical practice, and no objective tool is considered the golden standard. Future studies are planned to add objective assessments, such as pad testing and urodynamic evaluations, to better establish the clinical relevance of nerve conduction disorders. The strength of this research will help clarify whether the nerve conduction changes result from PFM dysfunction and LBP By using EMG assessment, it is an objective tool.

## Conclusion

These findings reveal statistically significant nerve conduction abnormalities in tibial, peroneal, and sural nerve conduction in women suffering from SUI and non-specific low back pain. The tibial nerve displayed extended distal and proximal latencies, diminished amplitudes, and lower nerve conduction velocity (NCV). The peroneal nerve showed prolonged distal latency and a reduced NCV, while the sural nerve had an extended onset latency, decreased amplitude, and slower NCV.

## Data Availability

Data will be held with the research author and may be available upon request from the corresponding author (Doaa A. Abdel Hady).
